# Obesity in Botswana: time for new cut-off points for abdominal girth?

**DOI:** 10.5830/CVJA-2016-060

**Published:** 2017

**Authors:** Churchill Lukwiya Onen,

**Affiliations:** Centre for Chronic Diseases, Gaborone, Botswana

**Keywords:** Botswana,, obesity,, waist circumference,, cut-off points,, modelling

## Abstract

**Introduction:**

Country-specific cut-off points for defining central obesity in black Africans are long overdue.

**Methods:**

Anthropometric data from 215 (51.4%) male and 203 (48.6%) female patients seen in Gaborone between 2005 and 2015 were analysed to establish appropriate cut-off points for waist circumference (WC) corresponding to a body mass index (BMI) of 30 kg/m^2^. Relative risks for cardiometabolic disorders were calculated for different BMI and WC categories using MedCalc®. The subjects’ mean age was 50.0 ± 10.8 years and 80.6% were Batswana.

**Results:**

Only 7.2% of patients had a BMI < 25 kg/m^2^, 27.3% were overweight and 65.5% were obese; mean BMI was 34.9 ± 6.5 kg/m2 in the women versus 31.0 ± 4.9 kg/m2 in the men (p < 0.0001). New cut-off points of 98 cm in men and 85 cm in women emerged. Different weight and WC categories appeared not to confer increased relative risk of hypertension, dysglycaemia or dyslipidaemia.

**Conclusion:**

The proposed WC cut-off values, if validated, should set the pace for larger studies across sub-Saharan Africa.

## Introduction

Several small observational studies in Botswana have produced inconsistencies in the prevalence of the metabolic syndrome (MetS), partly because of variations in methodological approaches to measurements of waist circumference and differences in study populations.^01^-^3^ Although Botswana was one of the poorest countries at independence, its diamond-dependent economy has propelled it to upper-middle income, with one of the fastestgrowing economies in the world, gross domestic product of $18 825 per capita in 2015, the fourth largest gross national income, and the highest human development index in sub-Saharan Africa.^4^,^5^

It is currently estimated that 57% of the population is urbanised. Overweight and obesity are therefore assuming epidemic proportions in the country. Life expectancy at birth is 63 and 65 years in men and women, respectively.^6^,^7^ This represents a 14-year increase for both genders between 2000 and 2012. The probability of dying between the ages of 15 and 60 years in men and women is 321 and 254 per thousand of the population, respectively.

In 2012, HIV/AIDS accounted for a third of the causes of mortality (5 700 deaths), whereas stroke, ischaemic heart disease, diabetes mellitus and hypertensive heart disease together accounted for about 15% of deaths (2 900 deaths). Cardiovascular diseases and diabetes together constituted the third most common cause of disability-adjusted life years (DALYs).

Since its description by Jean Vague8 nearly seven decades ago, abdominal obesity consistently features among criteria for the definition of the MetS, although the clustering of cardiovascular risk factors has greatly expanded. Obesity is also the bedrock in the International Diabetes Federation (IDF) definition of the MetS.^9^ The Joint Interim Statement (JIS) on the MetS recommended the use of population- and country-specific cut-off points to define an enlarged waist circumference.^10^ Accordingly, using non-validated cut-off points for waist circumference in the definition of obesity may falsify estimates of the MetS in the African setting. Inconsistent estimates of the MetS in sub-Saharan Africa have largely been due to lack of Africanspecific cut-off points for waist circumference.^11^-^13^

This study aimed firstly to determine the validity of current operational waist circumference cut-off points in Botswana; secondly, to determine the correlation between body mass index (BMI) and waist circumference (WC) in black African men and women, and in particular, the relationship between BMI of 30 kg/m2 and WC of 80 cm in women and 94 cm in men; and thirdly whether excessive body weight relates to cardiometabolic and other chronic medical disorders in the study population.

## Methods

Data from a heterogeneous group of adult patients seen over a 10-year period (2005–2015) at a specialised medical clinic I run in Gaborone city were extracted from conveniently sampled case notes, taking every sixth file from over 3 000 files accumulated in the filing room during a decade of private practice. Completeness of records was examined for the presence of weight (kg), height (cm), waist circumference (cm) and co-morbidities for each patient during the index visit.

From the inception of the clinic at Gaborone Private Hospital, anthropometric measurements have been routinely performed whenever possible, using standard methods. Weight (kg) and height (cm) were measured in a similar manner to the method described by Dowse and Zimmet,^14^ using a well-calibrated scale. BMI was derived by dividing weight (kg) by the square of height (m^2^). Able-bodied participants were instructed to stand upright with the back against the stand, heels together and eyes directed forward so that the top of the tragus of the ear was horizontal with the inferior orbital margin, and the measuring plate was lowered on to the scalp to give the correct height.

Waist circumference was measured with the individual standing upright with the side turned to the observer, who was often seated. A measuring tape attached to a spring, similar to that used in the INTERHEART study,^15^ was placed snugly in a horizontal plane around the subject’s abdomen, mid-way between the rib cage and the iliac crest, and standard tension was applied.

Demographic data, anthropometric measurements and co-morbidities for individual patients were entered into a database created using the Statistical Package for Social Sciences (IBM SPSS statistics 20®). Correlation plots were made for BMI (kg/m^2^) and WC (cm) in men and women, using 30 kg/m^2^ as the reference cut-off point for obesity, to determine corresponding mean WC (+ 95% CI) in both genders. Similar plots were made for BMI and WC using 94 cm in men and 80 cm in women as reference cut-off points for obesity to determine corresponding BMI (+ 95% CI) in both genders. Height (m) was also plotted against WC in both men and women.

Patients were grouped into five WHO weight categories:^16^ normal weight (category 1; BMI 18.5–24.9 kg/m^2^), overweight (category 2; BMI 25.1–29.9 kg/m^2^), grade I obesity (category 3; BMI 30.0–34.9 kg/m^2^), grade II obesity (category 4; BMI 35.0–39.9 kg/m^2^), and grade III obesity (category 5; BMI ≥ 40.0 kg/m^2^). Women were arbitrarily grouped into three WC categories: category 1 (WC ≤ 80 cm), category 2 (WC 80.0–87.9 cm) and category 3 (WC ≥ 88 cm). Men were likewise grouped into three WC categories: category 1 (WC ≤ 94 cm), category 2 (WC 94.0–101.9 cm) and category 3 (WC ≥ 102 cm).

## Statistical analysis

With MedCalc® software,^17^ using category 1 BMI and category 1 WC as references, relative risks (+ 95% CI) for hypertension, dysglycaemia and dyslipidaemia were calculated for different BMI and WC categories. Sample means and standard deviations were calculated in the conventional way. Level of statistical significance was taken to be p < 0.05.

## Results

A total of 498 case notes were retrieved; 23 did not contain the required data. Of 475 case notes of patients with the required anthropometric parameters, 20 naturalised non-black citizens of Botswana, 25 Asians and 12 Caucasians were excluded; the remaining 418 black African patients were analysed. This consisted of 215 men (51.4%) and 203 women (48.6%), mean age 50.0 ± 10.8 years, 80.6% of whom were Batswana and 19.4% were other black Africans.

Only 7.2% had normal weight (BMI 18.5–24.9 kg/m^2^), 27.3% were overweight (BMI 25–29.9 kg/m^2^) and 65.5% were obese (BMI > 30 kg/m^2^). Significantly more women were obese (77.8%) compared to men (54.0%); mean BMI was 34.9 ± 6.5 versus 31.0 ± 4.9 kg/m^2^ (p < 0.0001). Hypertension affected 77.8% (325/418) and dysglycaemia 44.3% (185/418) of the patients. Lipid profiles were not estimated in a third of the sample group. Dyslipidaemia was documented in 67% of the remaining 279 patients.

**Fig. 1.**
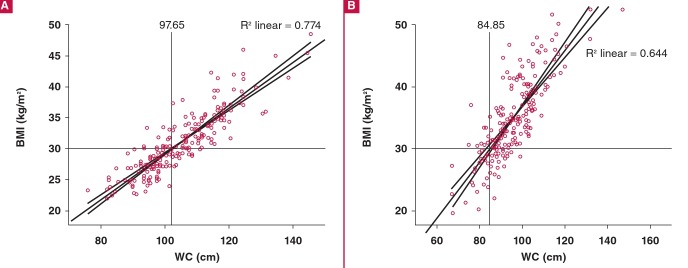
Correlation between BMI (kg/m^2^) and WC (cm) in (A) 214 men and (B) 203 women with BMI = 30 kg/m^2^ as cut-off point. BMI, body mass index; WC, waist circumference.

**Fig. 2.**
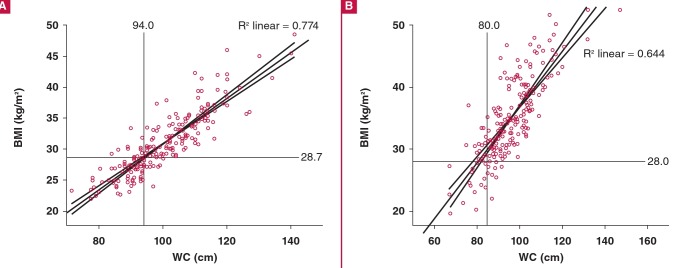
Correlation between BMI (kg/m^2^) and WC (cm) in (A) 214 men with WC = 94.0 cm and (B) 203 women with WC = 80 cm as cut-off point. BMI, body mass index; WC, waist circumference.

One man did not have a WC measurement and was excluded from the correlation plots. WC directly correlated with BMI in both genders (R^2^ linear = 0.774 in men; 0.644 in women) with new cut-off points of 98 cm (95% CI: 96.9–98.2 cm) in men and 85 cm (95% CI: 83.0–86.5 cm) in women, corresponding to BMI of 30 kg/m^2^. (Fig. 1A, B). The current operational WC of 94.0 cm in black African men corresponded to a BMI of 28.7 kg/m^2^, whereas in black women, the corresponding BMI was 28.0 kg/m^2^ for a WC of 80 cm (Fig. 2A, B).

**Fig. 3.**
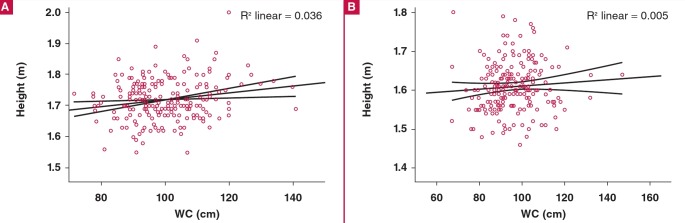
Poor correlation between height and WC in (A) 214 men and (B) 203 women. WC, waist circumference.

In both men and women, there was a poor correlation between height and WC (R^2^ linear = 0.036 in men; 0.005 in women) (Fig. 3A, 2B). There was no correlation between age and BMI among the 418 patients (R^2^ linear = 0.001).

Table 1 shows the relative risks of hypertension, dysglycaemia and dyslipidaemia for different BMI categories versus normal weight (BMI < 25 kg/m^2^) among 418 patients. Table 2 shows the relative risks of any cardiovascular disease for different WC categories versus current reference WC (< 80 cm in women; < 94 cm in men). Both tables demonstrate no overall statistically significant risk relationship with hypertension, dysglycaemia and dyslipidaemia. Separate analysis showed that WC ≥ 102 cm in men was associated with 21% increased total co-morbidity, combining cardiometabolic and musculoskeletal disorders (RR 1.21; 95% CI: 1.03–1.42; p = 0.022).

**Table 1:** Relative risks of hypertension, dysglycaemia and dyslipidaemia for different BMI categories versus normal weight (BMI < 25 kg/m2) among 418 patients

	(1) Hypertension, (2) dysglycaemia, (3) dyslipidaemia		
WHO BMI category (kg/m^2^)	Relative risk	95% CI	p-value
Overweight (25–29.9)	(1) 0.99	(0.78–1.27)	0.95
	(2) 0.94	(0.61–1.45)	0.78
	(3) 1.24	(0.79–1.96)	0.36
Grade I (30–34.9)	(1) 1.09	(0.87–1.38)	0.45
	(2) 0.88	(0.57–1.36)	0.57
	(3) 1.24	(0.79–1.95)	0.36
Grade II (35–39.9)	(1) 1.12	(0.88–1.43)	0.45
	(2) 1.01	(0.65–1.59)	0.95
	(3) 1.07	(0.66–1.74)	0.77
Grade III (> 40)	(1) 1.06	(0.82–1.38)	0.64
	(2) 1.02	(0.64–1.62)	0.94
	(3) 1.23	(0.76–1.98)	0.40

WHO, World Health Organisation; BMI, body mass index.

**Table 2:** Relative risks of any cardiovascular disease for different waist circumference categories versus current reference waist circumferences (< 80 cm in women; < 94 cm in men)

	Any CVD relative risk		
Waist circumference category (cm)	Relative risk	95% CI	p-value
Category 2			
Men (94–101.9)	1.04	(0.91–1.18)	0.61
Women (80–87.9)	1.15	(0.84–1.59)	0.39
Category 3			
Men (> 102)	1.10	(0.99–1.22)	0.08
Women (> 88)	1.17	(0.86–1.58)	0.32

CVD, cardiovascular disease refers to hypertension, dysglycaemia and dyslipidaemia.

## Discussion

There are strong indications for examining body size in the current context of affluence, social marketing and food consumption in Botswana. Anthropometric measurements are frequently used to determine parameters of overweight and obesity at most points in the healthcare system and during many ‘wellness’ programmes. Knowing that a person’s BMI exceeds 30 kg/m^2^ may be useful only in understanding the individual’s potential cardiometabolic risk and total burden of co-morbidity. After all, obesity may be an epiphenomenon for other cardiovascular disease risk factors. But failure to recognise obesity as a major health issue and its complex social and societal construct may camouflage the problem and propagate inherent imperfections of the obesity-screening processes.

Cataloguing BMI, WC and sometimes waist:hip ratios may not reflect their correlation to obesity-related sequelae. There are medically healthy obese individuals and metabolically obese normal-weight individuals, although the prevalence of these conditions in this community is unknown. National anthropometric data are scarce or unavailable.

The growing prevalence of overweight and obesity sweeping southern Africa, with a national prevalence between 30 and 60% of populations over the age of 15 years, is largely due to dietary shift away from high-fibre, low-calorie diets rich in fruits and vegetables towards refined, energy-dense foods high in fat, calories, sweeteners and salt, and this affects females disproportionately.^18^,^19^ A paradoxical situation, in which poverty and high levels of overweight and obesity co-exist in urban settings, may be explained by reduced levels of physical activity in all groups. Coupled with rapid urbanisation, industrialisation and increased sedentary lifestyles, these nutritional and demographic transitions have ushered in the rapid emergence of non-communicable diseases, including hypertension, diabetes, stroke, heart disease and other cardiovascular diseases.

Despite direct correlations between BMI and WC, findings from this situational analysis in Botswana suggest the need for new cut-off points for WC (98 cm in men; 85 cm in women) that correspond to a BMI of 30 kg/m^2^. Europid WC cut-off points (≥ 80 cm in women; ≥ 94 cm in men), as recommended by the IDF9 and currently used in sub-Saharan Africa to define central obesity do not appear to correlate with BMI ≥ 30 kg/m^2^ in Botswana. Elsewhere, there is a strong correlation between BMI of 25–34.^9^ kg/m^2^, WC ≥ 102 cm for men and ≥ 88 cm for women, and greater risk of hypertension, type 2 diabetes, dyslipidaemia and coronary heart disease.^20^

Western countries derived cut-off values of WC from correlation with BMI, whereas Asians tried to define WC cut-off values produced by receiver-operating characteristics (ROC) curve analysis.^21^,^22^ Measurements of skinfold thickness are less accurate, particularly in obese individuals and are therefore discouraged in routine screening exercises, except in epidemiological studies. Precise measurements of body fat using computed tomography (CT) or magnetic resonance imaging (MRI) scans or biochemical barometers such as adipokines are unlikely to be used outside research settings in Botswana. However, measurement of fasting insulin and glucose levels may help in the calculation of HOMA-IR in individuals with features of insulin resistance syndromes.

In the Diabetes and Macrovascular Complications study of 258 adult diabetic patients in Botswana,^1^ the MetS defined using IDF criteria^9^ was more prevalent in diabetic women compared to diabetic men. Depending on which set of parameters in the IDF criteria was used for the definition, the prevalence of the MetS ranged from 41.7–83.7% in men, and 37.8–88.6% in women. Obesity, defined by waist:hip ratio (> 0.9 in men, > 0.85 in women) was present in 87.9% of diabetics, and by WC (> 94 cm men, > 80 cm in women) in 79.0% of diabetics, but prevalence of the MetS dropped to 38.3% using BMI (> 30 kg/m^2^). Large disparities in estimates of the MetS based on different parameters complicated its true prevalence estimates in that study. BMI was viewed as an insensitive indicator of the MetS, especially in diabetic women.

Garrido et al.^2^ conducted a small cross-sectional, observational study of 150 hospital workers at a peripheral facility in Botswana, representing nearly half of the hospital workforce, women comprising over 70% of the group. The investigators applied any three or more of the ATP III criteria for definition of the MetS.^23^ Low high-density lipoprotein (HDL) cholesterol affected 80% of the group, dysglycaemia 73.3%, hypertension 44%, central obesity 42% and hypertriglyceridaemia 14%. A third of the participants met the ATP III criteria for the MetS and 28.7% had a BMI > 30 kg/m^2^. That over 40% of hospital employees had central obesity, using higher cut-off points for WC raises the possibility of a high prevalence of abdominal obesity in the community.

Another cross-sectional study by Malangu3 looked at 190 adult HIV-infected patients on highly active antiretroviral therapy (HAART) at Princess Marina Hospital in Gaborone in 2010. Their mean age was 42 ± 9.04 years and nearly threequarters of the group were women (74.2%). Using IDF criteria, the investigator showed an overall prevalence of the MetS in 11.1% of participants. Risk factors for the MetS included increased age, male gender and longer exposure to antiretroviral drugs, particularly protease inhibitors. Only 10% of participants had a BMI > 30 kg/m^2^, 13 of 141 women and eight of 49 men had abdominal obesity (WC ≥ 80 cm in women and ≥ 94 cm in men).

The study design lacked comparator control groups (e.g. non-HIV-infected individuals or HIV-infected persons pre-HAART), making it difficult to determine the independent contribution of antiretroviral therapy to the MetS and this limits generalisability of the findings. However, it appears that obesity and the MetS were substantially lower in HIV-infected individuals, despite the use of different diagnostic criteria for the MetS.

Studies from other parts of sub-Saharan Africa have generated wide variations in WC cut-off points. For example, central obesity defined by WC > 102 cm in men and > 88 cm in women was more common than generalised obesity (BMI > 30 kg/m^2^) in Cotonou, Benin.^11^ In South Africa, Motala et al.^12^ found that WC of > 86 cm in men and > 92 cm in women predicted the presence of at least two elements of the MetS in a cross-sectional, population-based study in a rural setting. That study was heavily gender biased, with 80% of the 947 participants being female.

In 2014 Magalhães et al.,^13^ in another cross-sectional study of 615 university employees in Luanda, Angola, found overall prevalence of overweight to be 47.8%, and obesity in 45.2% of participants. Using JIS criteria, crude and age-standardised prevalence of the MetS were 27.8 and 14.1%, respectively. The crude and age-standardised prevalence of the MetS was 17.6 and 8.7% using ATP III criteria,^23^ which apply higher WC cut-off points (≥ 102 cm in men, ≥ 88 cm in women).

Applying ROC curves of WC to detect the MetS, new cut-off points of this study were 87.5 cm in men (sensitivity 75.9%, specificity 81.2%) and 80.5 cm in women (sensitivity 88.4%, specificity 60.5%). The three most common criteria for the MetS were increased WC, hypertension and low serum HDL cholesterol levels. Women showed a higher prevalence in all age groups from the age of 30 years.

The INTERHEART study, a case-controlled study of 27 000 participants from 52 countries, showed a graded and highly significant association between waist:hip ratios (WHR) and acute myocardial infarction worldwide.^15^ The association of WHR with acute myocardial infarction in the INTERHEART study addressed one of the most fundamental cardiovascular sequelae of excessive and disproportionate weight. Although the INTERHEART study investigators cast doubt on the use of BMI in the context of acute myocardial infarction, obesity, however defined, was associated with a myriad of conditions, including hypertension, diabetes mellitus, dyslipidaemia, obstructive sleep apnoea, gastro-oesophageal reflux, sudden death, stroke, certain types of cancer, infertility, degenerative joint disease and negative psychosocial impact.

The Prospective Studies Collaboration addressed the association of BMI with cause-specific mortality in about 900 000 adults in 57 prospective studies.^24^ These authors concluded that other anthropometric measures such as WC and WHR could well add extra information to BMI, and BMI to them, but that BMI is in itself a strong predictor of overall mortality rate both above and below the apparent optimum of about 22.5 to 25 kg/m^2^.

For screening purposes, it appears that measurements of WHR provide no advantage over WC alone, are cumbersome and may be fraught with errors in field situations. Furthermore, it may not be necessary to measure WC in persons with BMI > 35 kg/m^2^ since it adds little value in the predictive power of disease-risk classification.^25^ Inconsistencies in cut-off values for WC have potentially undesirable consequences for cardiovascular risk stratification, disease categorisation and prioritisation of preventative strategies for obesity. There is therefore a strong need for validation of these WC cut-off values for Botswana before they can be used for prediction of incident outcomes such as cardiovascular diseases or type 2 diabetes mellitus.

Modelling may help to capture the scope and complexity of the obesity problem in Botswana. Applications of heterogeneous adaptive pieces of the puzzle that are affected by and/or influence the overall behaviour of individuals within society may lead to the development of empirically based public health models. Agent-based modelling (ABM) represents one such simplified example.^26^ Using the ABM approach, agents could represent individuals, their attributes, behaviours and relationships with other individuals in society. The environment could represent geographical locations, mobility, domestic settings, market forces and social networking.

Systematic dynamic modelling (SDM) or perhaps more appropriately for Botswana, the MicroSimulation model, could be used to establish temporal and causal associations, if any, between obesity and related disorders, such as hypertension, diabetes, abnormal lipids, cardiovascular diseases, cancers, degenerative musculoskeletal disorders and psychological afflictions.^27^ The strategy focuses on ‘upstream’ preventive approaches rather than ‘downstream’ acute and chronic care. The goal is to enhance the number of safer, healthier people and prevent others from becoming vulnerable or being afflicted by obesity and its related complications.

There are, however, several limitations of this study worth mentioning. Firstly, this was a retrospective analysis of case notes of a small number of patients seen at a specialised private medical practice. The finding may not therefore apply to the general population. Secondly, WC reflects both subcutaneous and visceral fat and at best represents a crude surrogate for visceral adiposity. Because women generally have more subcutaneous fat, there is a potential risk of misclassifying them as viscerally obese, thereby resulting in overestimation of the MetS in women. Thirdly, little is known about the full impact of the obesity epidemic on the health of the community, and failure to demonstrate statistically significant links between obesity and existing co-morbidities in this study should not be construed to suggest benigness of obesity in this population.

## Conclusion

This study reiterates the need for ethnic-specific WC cut-off points for defining central obesity and, by extension, for diagnosis of the MetS among black Africans. The proposed WC cut-off values, if validated, will set the pace for larger studies across sub-Saharan Africa. Variations in WC cut-off values illustrate the uniqueness of populations.
